# Why do earlier‐arriving migratory birds have better breeding success?

**DOI:** 10.1002/ece3.5441

**Published:** 2019-07-19

**Authors:** Catriona A. Morrison, José A. Alves, Tómas G. Gunnarsson, Böðvar Þórisson, Jennifer A. Gill

**Affiliations:** ^1^ School of Biological Sciences University of East Anglia Norwich UK; ^2^ Department of Biologia & CESAM – Centre for Environmental and Marine Studies University of Aveiro Aveiro Portugal; ^3^ South Iceland Research Centre University of Iceland Laugarvatn Iceland

**Keywords:** arrival dates, demography, laying dates, migration, phenology, productivity, reproductive success

## Abstract

In migratory birds, early arrival on breeding sites is typically associated with greater breeding success, but the mechanisms driving these benefits are rarely known. One mechanism through which greater breeding success among early arrivers can potentially be achieved is the increased time available for replacement clutches following nest loss. However, the contribution of replacement clutches to breeding success will depend on seasonal variation in nest survival rates, and the consequences for juvenile recruitment of hatching at different times in the season. In particular, lower recruitment rates of late‐hatched chicks could offset the benefits to early arrivers of being able to lay replacement clutches, which would reduce the likelihood of replacement clutch opportunities influencing selection on migratory timings. Using a simulation model of time‐constrained capacity for replacement clutches, paramaterized with empirically‐derived estimates from avian migratory systems, we show that greater reproductive success among early‐arriving individuals can arise solely through the greater time capacity for replacement clutches among early arrivers, even when later renesting attempts contribute fewer recruits to the population. However, these relationships vary depending on the seasonal pattern of nest survival. The benefits of early arrival are greatest when nest survival rates are constant or decline seasonally, and early arrival is least beneficial when nest success rates increase over the breeding season, although replacement clutches can mitigate this effect. The time benefits of early arrival facilitating replacement clutches following nest loss may therefore be an important but overlooked source of selection on migratory timings. Empirical measures of seasonal variation in nest survival, renesting, and juvenile recruitment rates are therefore needed in order to identify the costs and benefits associated with individual migration phenology, the selection pressures influencing migratory timings, and the implications for ongoing shifts in migration and breeding phenology.

## 1 INTRODUCTION

For migratory species, the timing of migratory journeys can have important fitness consequences. Declines in breeding success with date of arrival on the breeding grounds have been widely demonstrated in many species (Aebischer, Perrin, Krieg, Studer, & Meyer, 1996; Currie, Thompson, & Burke, 2000; McKellar, Marra, & Ratcliffe, 2013; Norris, Marra, Kyser, Sherry, & Ratcliffe, 2004; Rockwell, Bocetti, & Marra, 2012; Saino et al., 2004; Sergio, Blas, Forero, Donázar, & Hiraldo, 2007; Velmala et al., 2015), and a range of mechanisms have been proposed to explain this association, but empirical evidence of the mechanisms driving arrival date‐breeding success links is still scarce. Declines in breeding success with arrival date could arise as a result of variation in individual capacity to both migrate early and breed successfully, irrespective of the conditions encountered during the breeding season. If better quality individuals both arrive first and have greater breeding success (Verhulst & Nilsson, 2008), for example because they are older (Daunt, Wanless, Harris, & Monaghan, 1999), have a greater capacity to attract a mate (Bensch & Hasselquist, 1992), lay larger clutches (Bêty, Gauthier, & Giroux, 2003) and/or provision and protect their offspring, then arrival date may be a correlate, rather than the driver, of breeding success, and selection pressure on arrival timings may be weak. Alternatively, greater breeding success among early arrivers could result from local environmental variation, with early arrivers potentially having more opportunities to occupy better quality habitats and territories (Currie et al., 2000; Harris, Heubeck, Shaw, & Okill, 2006; Jonzén, Hedenström, & Lundberg, 2006) in which, for example, eggs or chicks may be at lower risk of predation or greater resource availability may improve offspring growth and survival (Arnold, Hatch, & Nisbet, 2004). However, breeding dispersal events are relatively rare (Paradis, Baillie, Sutherland, & Gregory, 1998), suggesting that any association between arrival timing and site quality must arise in the year of recruitment and persist thereafter. An alternative, and often neglected, driver of associations between migratory timings and breeding success is variability in the time available for replacement clutches following nest loss, or for rearing multiple broods (Hoffmann, Postma, & Schaub, 2015; Saino et al., 2004). If the fitness benefits of early arrival operate primarily through the time available for replacement clutches, then the advances in spring migration that are currently occurring in many species (Knudsen et al., 2011; Rubolini, Møller, Rainio, & Lehikoinen, 2007) could have profound implications for both productivity and the phenology of successful nests. Such changes could be contributing to current divergent population trajectories in migratory species with differing rates of advancing spring migration (Gilroy, Gill, Butchart, Jones, & Franco, 2016; Møller, Rubolini, & Lehikoinen, 2008), and altering the benefits of early arrival.

Among migratory species, having sufficient time to rear multiple broods can be rare but rates of nest loss as a consequence of predation, severe weather or human actions are often high, and replacement clutches are common in migratory bird species (Newton, 2008) and occur even at high arctic latitudes (Jamieson, 2011; Johnson et al., 2011). Most bird species are capable of renesting (Martin, 1995; Thompson, Knadle, Brubaker, & Brubaker, 2001); however, the probability of replacement clutches being laid following nest loss typically declines seasonally (Brinkhof, Cavé, Daan, & Perdeck, 2002; Hansson, Bensch, & Hasselquist, 2000; Hipfner, Gaston, Martin, & Jones, 1999; Jamieson, 2011; Pakanen, Rönkä, Thomson, & Koivula, 2014; Weggler, 2006), suggesting that opportunities to renest will be greater for early‐nesting individuals. Given the constraints of postbreeding molt and migration, insufficient time to complete replacement clutches (and thus a lower probability of renesting) later in the breeding season is likely to be a feature common to all migratory species. The benefits of laying replacement clutches will be influenced by seasonal variation in both (a) the probability of nest survival and (b) the probability of successful subsequent recruitment of offspring. Seasonal variation in nest success rates can arise through seasonal variation in factors such as local predator abundance and activity (DeGregorio, Weatherhead, Ward, & Sperry, 2016; Sperry, Peak, Cimprich, & Weatherhead, 2008), weather conditions (Skagen & Adams, 2012) and nest concealment (Borgmann, Conway, & Morrison, 2013). Seasonal declines in offspring recruitment probability have been reported in many species (Alves, Gunnarsson, Sutherland, Potts, & Gill, 2019; Clark, Pöysä, Runko, & Paasivaara, 2014; Harris, Buckland, Russell, & Wanless, 1994; Lok, Veldhoen, Overdijk, Tinbergen, & Piersma, 2017; Visser et al., 2015), potentially reflecting difficulties facing late‐hatched young in locating and amassing resources during the postfledging and winter periods. Lower recruitment of late‐hatched young would be likely to reduce the benefits associated with replacement clutches. Consequently, the contribution of replacement clutches to the benefits of early arrival will depend on breeding phenology (including the length of incubation and the time between nest loss and replacement), nest survival rates and offspring recruitment probabilities, and how these rates vary seasonally.

Here, we construct a simulation model to explore the relationships between timing of arrival and breeding success that can arise through variation in the time available to lay replacement clutches. We then explore how these relationships vary with seasonal variation in nest survival and the consequences for offspring recruitment.

## 2 MATERIALS AND METHODS

### 2.1 Simulation model

**Figure 1:**
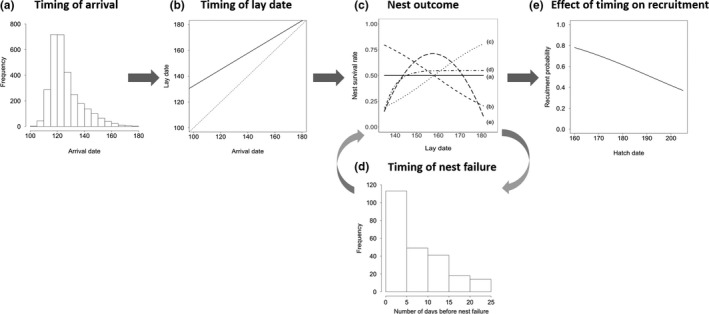
Model structure and the distributions and functions used in the simulations: (a) distribution of individual arrival dates, (b) the relationship between individual arrival date and lay date (solid line; see Equation S1) and the gap between individual arrival and laying (vertical distance between the line of unity (dashed line) and the solid line), (c) the five scenarios of seasonal variation in nest survival rates (details in text), (d) the distribution of days between lay date and nest failure and (e) the relationship between hatch date and the probability of offspring subsequently recruiting into the breeding population (see Equation S2)

We constructed a simulation model in R 3.3.1 (R Core Development Team, 2016) of 1,000 individuals with differing timings of arrival on the breeding grounds. Fifteen simulations were conducted, with each being assigned a maximum number of replacement clutches (either zero, one or three) and one of five different scenarios of seasonality of nest survival rates (see below), and each simulation was run 100 times. In all simulations, each individual could complete a maximum of one successful nesting attempt (i.e., nest successfully hatched) per year, over a 10‐year period. To ensure that biologically realistic values were used in the simulations, the modeled distributions and functions (see Figure 1) were constructed using data from published studies of breeding waders in Iceland (Alves et al., 2019; Þórisson, 2013), but the key features of the model (individual variation in arrival dates, seasonal variation in nest survival, replacement clutch opportunities and offspring recruitment probabilities) are applicable to all migratory bird systems.

### 2.2 Estimating timing of arrival and laying of first clutch

Individuals were assigned an arrival date by randomly sampling from a left‐skewed arrival distribution (Figure 1a and SOM 1.1), and the assigned arrival dates for each individual were then fixed for the 10‐year period over which simulations were run, as individuals typically show repeatability in their spring arrival dates (Gill et al., 2014). For each individual, timing of nesting (lay date) was then calculated as a fixed function of arrival dates (Figure 1b and SOM 1.2), with a slightly larger gap (+29 days) between arrival and laying at the start of the season, reflecting the greater likelihood of weather constraints on nesting at the start of the season.

### 2.3 Predicting nest survival rates

In order to explore how the influence of renesting on nesting success might vary with nest survival rates, we first modeled nine different levels of nest survival rate (from 0.1 to 0.9) which were constant throughout the season. As these models showed that the effects of renesting were greatest at intermediate levels of nest survival (see Section 3), we then explored five realistic scenarios (see Section 4) of seasonal variation in nest survival (Figure 1c), all of which had an intermediate mean rate of nest survival (0.5): (a) constant nest survival throughout the season, (b) sustained seasonal decrease, (c) sustained seasonal increase, (d) seasonal increase to an asymptote, and (e) unimodally distributed nest survival, see SOM section 1.3 for details. The success (hatch or fail) of each nesting attempt was determined by a random draw from a binomial distribution, with the probability of success equal to the nest survival rate predicted from the lay date of each nesting attempt in each nest survival scenario.

### 2.4 Modeling replacement clutch occurrence and timing

In simulations in which replacement clutches were possible (up to a maximum of one or three attempts), the lay date of renesting attempts was determined by first assigning a nest failure date to each failed attempt, by sampling at random from a distribution of numbers of days between laying and failure of nesting attempts (Figure 1d), and adding a fixed gap between nest failure and renesting of four days (renesting gap, Þórisson, 2013). This process was continued until either a nesting attempt was successful, the maximum number of renesting attempts was reached (one or three) or lay‐dates exceeded the end of the breeding season on day 181 (last day on which nesting attempts could be initiated, Þórisson, 2013).

### 2.5 Estimating seasonal variation in recruitment probabilities

The probability of offspring recruitment into the breeding population for each successful nesting attempt (one hatched offspring per successful attempt) was estimated as a function of hatch date (Figure 1e, SOM 1.3, Alves et al., 2019). Hatch dates were estimated to be 25 days after the laying date of the successful nesting attempt. The outcome of each recruitment event was then determined by a random draw from a binomial distribution with probability of success equal to the recruitment probability. In the cases where a successful nesting attempt did not take place during that breeding season, recruitment probability was set to zero.

Each of the 15 simulations was run 100 times, from which the arrival date, mean lay date of successful nests, mean annual number of nesting attempts, mean annual number of successful nesting attempts, mean annual recruitment probability and lifetime number of recruits over the 10‐year period was calculated for each individual.

## 3 RESULTS

### 3.1 Replacement clutch capacity, nest survival rates, and breeding success

**Figure 2:**
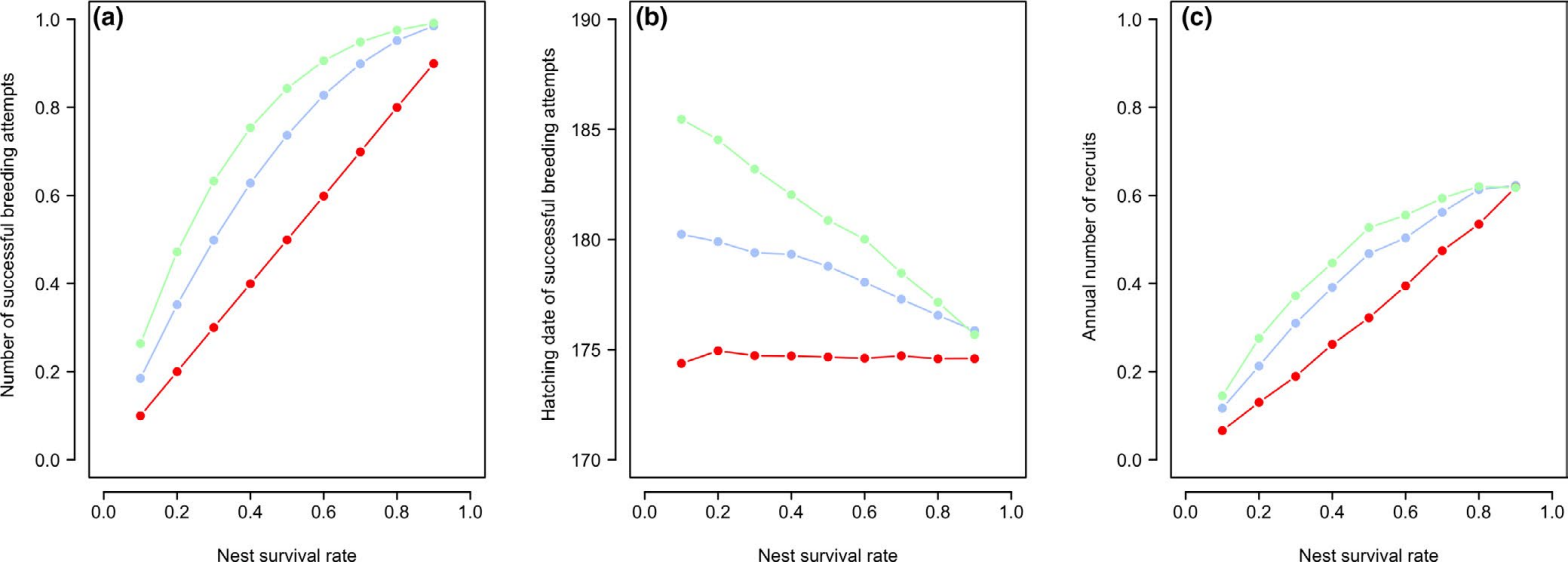
Simulated effects of differing nest survival rates on the (a) number of successful nesting attempts, (b) hatching date of successful nesting attempts and (c) annual number of recruits for differing maximum numbers of possible replacement clutches following nest loss (red = zero, blue = one, green = three)

As nest survival rates increase, the number of successful nesting attempts increases, and the capacity to lay multiple replacement clutches results in a higher number of successful nesting attempts (Figure 2a). The increase in the number of successful nesting attempts is most rapid in populations that have the capacity to lay replacement clutches; however, this increase slows at higher rates of nest survival, as the success of first nests renders replacement clutches increasingly redundant (Figure 2a). Low nest survival rates also result in later average hatch dates of successful nesting attempts when replacement clutches are possible (Figure 2b), as a greater proportion of successful attempts are from replacement clutches. Consequently, the correlation between arrival‐ and lay‐dates is weakened when replacement clutches are possible (Figure S1). The mean annual number of recruits also increases with nest survival rate and is highest in populations that can lay replacement clutches (Figure 2c), but this benefit diminishes at high nest survival rates, again because of high success of first nests in all populations renders replacement clutches increasingly redundant.

### 3.2 Seasonal variation in nest survival rates

**Figure 3:**
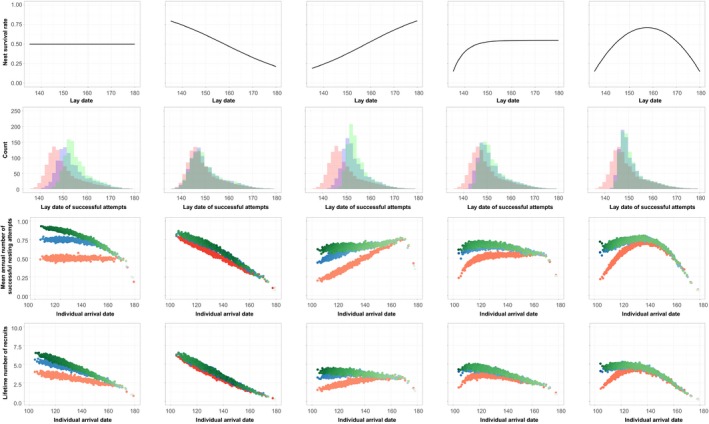
Variation in the consequences of individual spring arrival dates for the probability of successful nesting and offspring recruitment, in simulation models with differing seasonal patterns of nest survival (top row) and maximum number of possible replacement clutches (red = zero, blue = one, green = three). For each seasonal nest survival scenario, the distribution of lay‐dates of successful nesting attempts (second row), relationships between mean annual arrival date and both number of successful nesting attempts (third row) and lifetime number of recruits (fourth row) are shown (see Figure 1 and SOM for model details). Color intensity (rows 3 and 4) represents the average annual number of nesting attempts (darker = more nesting attempts [range: 0.5–2.5])

Five scenarios of seasonal variation in nest survival rates were modeled (Figure 3, top row). When nest survival rates are constant throughout the breeding season (Figure 3, first column), the capacity for multiple replacement clutches results in a shift in lay‐dates of successful nests to later in the season (Figure 3b), more successful nesting attempts overall and a steeper decline in annual number of successful nesting attempts with arrival date (Figure 3c). Thus, with constant nest survival rates, the capacity to lay replacement clutches can generate strong relationships between arrival date and nesting success while, if replacement clutches are not possible, nesting success varies little with arrival date (Figure 3c). However, the benefits of replacement clutches can be greatly reduced if the offspring of late nests are less likely to recruit into the adult population. Consequently, seasonal declines in offspring recruitment probabilities reduce the impact of replacement clutches on the lifetime number of recruits, but early arrivers still achieve higher numbers of recruits overall (Figure 3d). Thus, when nest survival rates are constant and replacement clutches are possible, early arrival can facilitate a higher probability of achieving a successful nesting attempt, and these benefits of early arrival can persist even if seasonal declines in recruitment reduce the success of replacement clutches later in the season. These patterns persist when variation in individual arrival dates is introduced (Figure S2).

Seasonal variation in nest survival rates can alter the influence of replacement clutch capacity on breeding phenology and success (Figure 3e–t). Seasonal declines in nest survival (Figure 3e) result in a similar distribution of lay‐dates of successful nests across all three renesting frequencies (Figure 3f), as replacement clutches are only likely to be successful when they occur very early in the season. Consequently, the mean number of successful nesting attempts declines strongly with arrival date, but the capacity to lay replacement clutches only slightly increases the number of successful nesting attempts (Figure 3g) and the lifetime number of recruits achieved (Figure 3h).

Sustained seasonal increases in nest survival rates (Figure 3i) can introduce costs of arriving early, particularly when replacement clutches do not occur following loss of early nests. In this scenario, the majority of successful replacement clutches occur later in the season (Figure 3j), and thus, the mean annual number of successful nesting attempts varies little with arrival date when replacement clutches are possible and increases with arrival date when only single nesting attempts are possible (Figure 3k). However, seasonal declines in recruitment probability can offset benefits of arriving or renesting later in the season such that early‐arriving renesters can achieve more lifetime recruits (Figure 3i).

When nest survival rates are low at the start of the season and either increase to a plateau (Figure 3m) or decrease after peaking in mid‐season (Figure 3q), replacement clutches can again result in more successful nests but with slightly later laying dates (Figure 3n,r). Replacement clutches can mitigate the low nest survival in the early season such that the mean number of successful nesting attempts is lowest for late‐arrivers in both scenarios (Figure 3o,s), and seasonal declines in recruitment probability can further enhance the benefits of early arrival and replacement nest capacity for the lifetime number of recruits (Figure 3p,t).

Thus, replacement clutches can drive benefits of early arrival even when patterns of nest survival vary seasonally, and seasonal declines in recruitment probability can enhance benefits of early arrival and replacement clutches, even when nest survival rates increase through the breeding season.

## 4 DISCUSSION

Declines in breeding success with date of arrival date on the breeding grounds have been widely reported, and a range of potential drivers have been proposed (Currie et al., 2000; Gunnarsson, 2006; Møller, 1994). Our simulation models demonstrate that these patterns can be generated solely by early‐arriving individuals having more time to lay replacement clutches, even when recruitment probabilities are lower for later‐hatched offspring. Our models also show that replacement clutches are likely to be most beneficial at intermediate nest survival rates (Figure 2), and that early arrival and the capacity to lay replacement clutches can potentially increase the total number of offspring recruited, even if nest survival rates increase during the breeding season (Figure 3). Understanding the extent to which the time available for replacement clutches is driving the widely observed declines in breeding success with spring arrival date of migratory birds will therefore require empirical measures of replacement clutch rates and seasonal variation in nest survival and recruitment rates.

The benefits of replacement clutches for enhancing the probability of achieving a successful nesting attempt are most apparent when nest survival rates are at intermediate levels, as very high nest survival rates inevitably lead to the majority of first broods being successful (and thus limited benefits of renesting), while very low nest survival rates lead to the majority of first nests and replacement clutches failing (again limiting the benefit of renesting). Our models did not include opportunities to achieve more than one successful nesting attempt within a season but producing multiple broods would be likely to produce similar benefits to early arrival as laying replacement clutches. The probability of laying replacement clutches is also likely to be influenced by length of the breeding season and consequently to vary with latitude. However, replacement clutches can occur even at high arctic latitudes (Jamieson, 2011; Johnson et al., 2011), and thus, the benefits of early arrival may be particularly strong at higher latitudes, where breeding seasons are very short. Finally, replacement clutches could also incur costs for parents (Wendeln, Becker, & González‐Solís, 2000), which may influence the frequency with which replacement clutches are laid even if sufficient time is available.

Nest survival rates in wild bird populations can show seasonal declines (Grant, Shaffer, Madden, & Pietz, 2005; Sandercock, 1998; Weiser et al., 2018), increases (Reneerkens et al., 2016; Wilson, Martin, & Hannon, 2007), mid‐season peaks (Sperry et al., 2008) or little seasonal variation in survival (Laidlaw, Smart, Smart, & Gill, 2015; Sandercock, 1998; Weiser et al., 2018), and these patterns may vary spatially and between years as a result of differences in weather conditions, predator abundance and breeding phenology, availability of alternative prey for predators, etc. However, declines in the number of successful nesting attempts with individual arrival date were apparent in all of the seasonal nest survival scenarios modeled here, except for sustained seasonal increases in nest survival (Figure 3k). In addition, the apparent benefits of late arrival in systems with sustained seasonal increases in nest survival were reduced by renesting capacity (Figure 3k) and reversed by additional seasonal declines in subsequent life time number of recruits (Figure 3l). Thus, benefits of early arrival are likely to be apparent in most scenarios of seasonal variation in nest survival rates, and having the time to lay replacement clutches can be a major driver of these benefits.

Seasonal declines in recruitment probability can substantially increase the benefits associated with early arrival and nesting in migrants, even when (later) replacement clutches delay offspring fledging (Figure 3). Quantifying the recruitment probabilities of individuals that hatch and fledge at different points in the season requires long‐term tracking of individuals from the first year of life. Developments in tracking technologies have facilitated an increase in the number of studies capable of generating such data, and seasonal declines in recruitment probabilities are commonly reported in these studies (Alves et al., 2019; Clark et al., 2014; Lok et al., 2017; Visser et al., 2015). The mechanisms underpinning the costs of late fledging are likely to include the having less time available to locate resources and suitable wintering locations and, potentially, having fewer opportunities to gain social information from adults which may have already departed for winter sites (Gunnarsson, Gill, Newton, Potts, & Sutherland, 2005), and density‐dependent costs associated with fledging into local populations comprising large numbers of fledglings from earlier nests (Verhulst, 1992). More studies of seasonal variation in individual recruitment rates in migratory species will help to identify the magnitude of these effects and their drivers.

A striking feature of migratory populations is that, despite the apparent benefits of early arrival on the breeding grounds, the timing of spring migration within populations is typically characterized by high levels of between‐individual variation (e.g., arrival may span several weeks) but very low levels of within‐individual variation (individuals are typically highly repeatable in their timing of migratory journeys; Alerstam, Hake, & Kjellén, 2006; Brodersen, Ådahl, Brönmark, & Hansson, 2008; Conklin, Battley, & Potter, 2013; Gill et al., 2014; Phillips, Silk, Croxall, Afanasyev, & Bennett, 2005; Tibblin, Forsman, Borger, & Larsson, 2016). This suggests that the benefits of individual consistency in timing may be greater than the benefits of early arrival per se, and/or that the benefits of early arrival may be offset by costs that are not distributed equally among individuals. Previous models of migratory timings have assumed the latter, by incorporating variation in individual quality that directly influences timing of arrival (Kokko, 1999; Kokko, Gunnarsson, Morrell, & Gill, 2006). However, between‐individual variation in arrival dates could also arise through factors such as conditions in the year of recruitment (e.g., weather or individual condition) influencing individual timings, and benefits of consistency in individual timings could subsequently maintain this variation independent of any consistent variation in individual quality. A recent study demonstrated that population‐level shifts in spring arrival dates were driven by increases in the frequency of early‐arriving recruits in the population, and not by individuals altering arrival dates (Gill et al., 2014), suggesting that factors operating prior to recruitment influence individual arrival dates, which are then repeated in subsequent years. Migratory birds are often highly mate‐faithful between years, and studies have shown high levels of synchrony in arrival of mates (Fayet, Shoji, Freeman, Perrins, & Guilford, 2017; Gunnarsson, Gill, Sigurbjörnsson, & Sutherland, 2004; Phillips et al., 2005). Costs of later arrival may therefore be reduced by synchronous arrival of mates facilitating breeding soon after arrival, and the importance of synchronous arrival may underpin the benefits of consistent individual arrival timings.

## 5 SUMMARY

In summary, early arrival of migratory birds on breeding grounds can potentially lead to higher reproductive success solely as a result of the greater time available for laying replacement clutches, should early nesting attempts fail. These patterns persist across a range of seasonal patterns in nest survival rates and even when later nesting attempts are less likely to produce successful recruits. These benefits of replacement clutches are most apparent at intermediate nest survival rates, as very high or very low rates of nest loss will render replacement clutches unsuccessful or unnecessary, respectively. Advances in the timing of spring migration are occurring in many species at present, and there is evidence for population declines being associated with a lack of these shifts in timing (Gilroy et al., 2016; Møller et al., 2008). Our findings highlight the potential role of replacement nests as a driver of these divergent population trends; advances in spring migration could result in increased time available for replacement nests following nest loss, but the benefits of these replacement nests (and thus their potential contribution to population growth) will depend on the seasonal variation in nest survival and offspring recruitment. Empirical studies of the frequency and seasonality of replacement clutches are therefore urgently needed in order to identify the conditions in which they occur and their role as a driver of both the benefits of early arrival and the population‐scale consequences of shifts in migration timing.

## CONFLICT OF INTEREST

None declared.

## AUTHOR CONTRIBUTIONS

All authors conceived the ideas and designed the methodology. CAM performed the simulation modeling and JAG and CAM led the writing of the manuscript. All authors contributed to the writing of the manuscript and gave final approval for publication.

## Data Availability

No original data are presented in this manuscript.
