# Supplementary material for: Why do earlier‐arriving migratory birds have better breeding success?
Source: Ecol Evol. 2019 Jul 19;9(15):8856–64. doi: 10.1002/ece3.5441 (PMC6686336; doi:10.1002/ece3.5441)
Supplement: Supplementary file 1 [file ECE3-9-8856-s001.docx]

Supplementary Material

**Why do earlier-arriving migratory birds have better breeding success?**

Catriona A. Morrison^1.^, José A. Alves^2,3^, Tómas G. Gunnarsson^3^, Böðvar Þórisson^3^ and Jennifer A. Gill^1.^

1. School of Biological Sciences, University of East Anglia, Norwich Research Park, Norwich NR4 7TJ, UK.
2. Dep. Biologia & CESAM – Centre for Environmental and Marine Studies, University of Aveiro, Campus Universitário de Santiago, 3180-193 Aveiro, Portugal.
3. South Iceland Research Centre, University of Iceland, Lindarbraut 4, IS-840 Laugarvatn, Iceland.

**
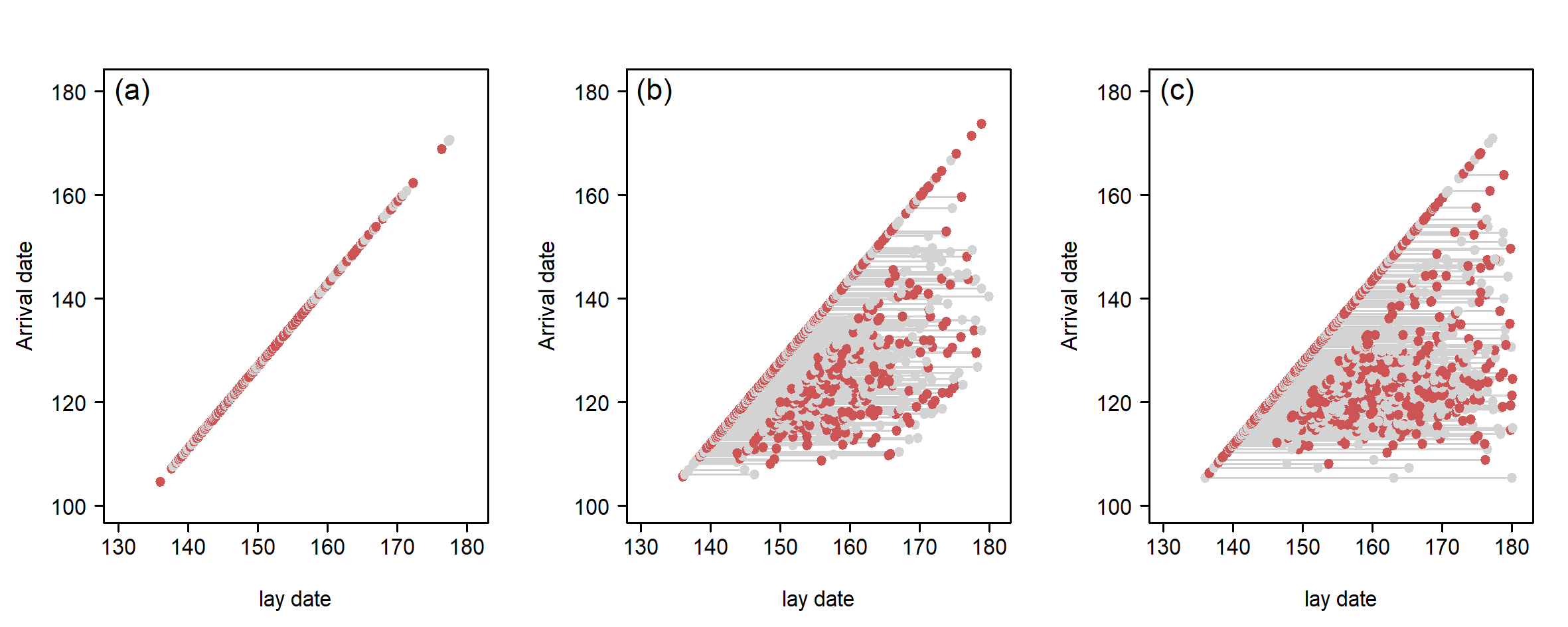
**

**Figure S1:** Lay dates for successful (grey) and unsuccessful (red) nests of individuals arriving at different times when (a) zero, (b) one and (c) three re-nesting attempts are possible. Grey lines connect each individual’s nesting attempts. Example from one iteration of the constant nest survival rate model (Figure 3a).


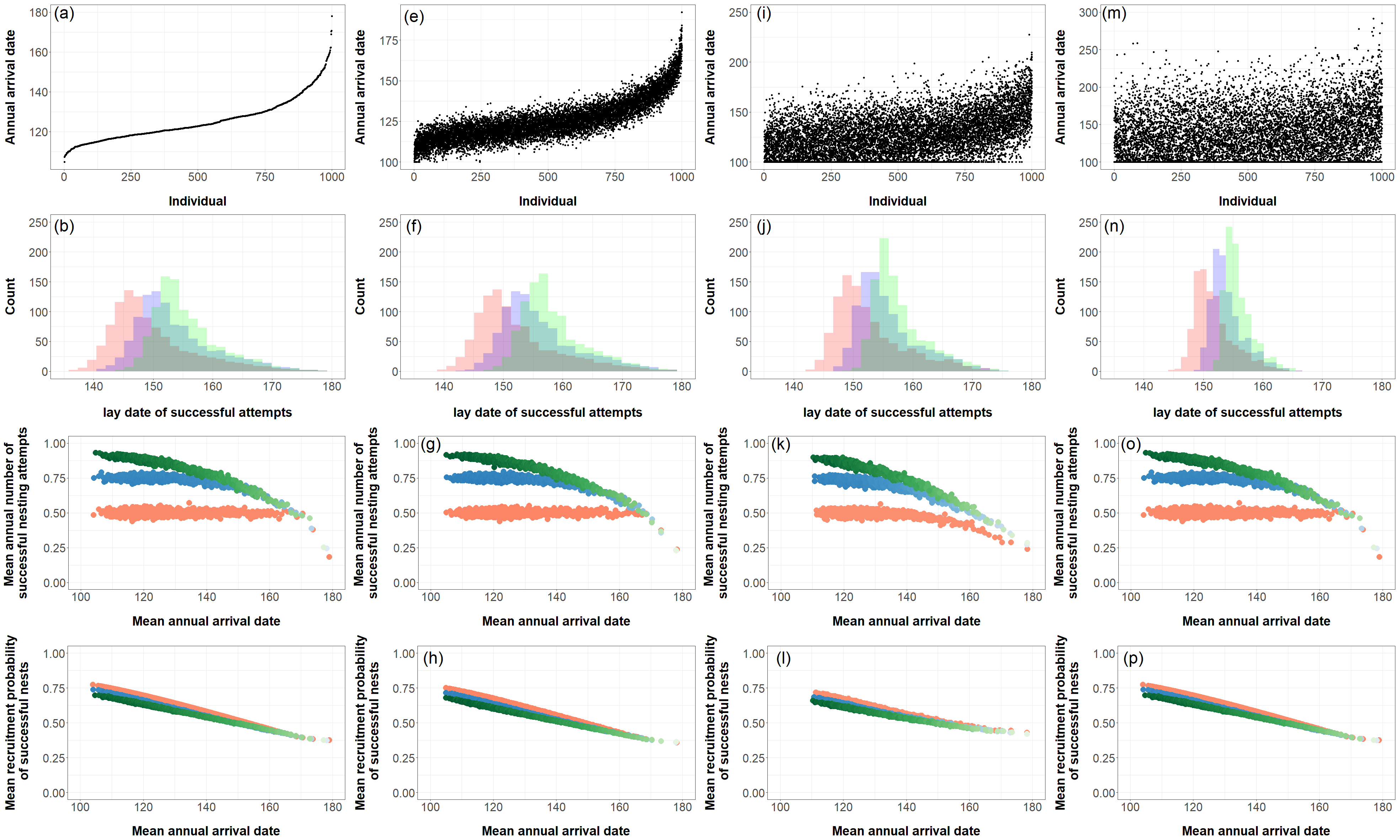


**Figure S2**: Variation in the consequences of individual spring arrival dates for the probability of successful nesting and offspring recruitment, in simulation models with differing levels of individual variation in arrival dates (top row) and maximum number of possible replacement clutches (red = zero, blue = one, green = three). Individual arrival date was allowed to vary by SD of (a) 0, (b) 5, (c) 20 and (d) 40 days. For each scenario, the distribution of lay dates of successful nesting attempts (second row), relationships between mean annual arrival date and both number of successful nesting attempts (third row) and lifetime number of recruits (fourth row) are shown. Colour intensity (rows 3 and 4) represents the average annual number of nesting attempts (darker = more nesting attempts (range: 0.5-2.5)).

Simulation model

*1.1 Generation of arrival distribution*

For 700 individuals, we sampled randomly from a normal distribution with a fixed mean (120) and standard deviation (5). For the remaining 300 individuals, we sampled from a skewed distribution, with a mean arrival date of 140, a standard deviation of 10 and a skewness parameter of 100. This resulted in a right-skewed distribution of arrival dates in each population of 1000 individuals (Figure 1a).

*1.2 Generation of lay dates*

Equation S1 was used to predict lay date as a function of arrival date:

**Equation S1:** Laying date = **69.496 + 0.63 *** arrival date (adapted from Þórisson, 2013)

*1.3 Generation of recruitment probabilities*

Equation S2 was used to predict a recruitment probability as a function of hatch date:

**^Equation^ S2:** logit(recruitment probability) = 2.27 -0.04 * hatch date (from Alves *et al.* 2019)

*1.4 Nest survival functions*

a) Constant nest survival throughout the season:

**Equation S3:** Nest survival rate = constant

Where constant = 0.5

b) Sustained seasonal decrease:

**Equation S4:** logit(nest survival rate) = constant+slope*lay date

Where constant = 1.36 and slope = 0.06

c) Sustained seasonal increase:

See equation S4:

Where constant = -1.44 and slope = 0.06

d) Seasonal increase to an asymptote:

**Equation S5:**

Nest survival rate = constant-a*exp(-b*lay date)

Where constant = 0.55, a = 0.4, b = 0.2

e) Unimodally distributed nest survival:

**Equation S6:**

Nest survival rate = constant + b*lay date + a*lay date^2^

Where constant = 0.15, a = -0.001, b = 0.05

*1.5 R code for simulation model:*

####Example code for running the simulation model with constant seasonal nest-survival rates and four re-nesting attempts.

###Load packages

library(fGarch)

library(faraway)

###

#data

###time to fail data

time.f<-read.csv('timetofaildata.csv') ###read in dataset

###Model specifications

yyears<-10

attempts<-4 ### number of possible re-nesting attempts allowed each season

###Parameters for nest survival

constant<-0.5

###laying date and arrival relationship

LD.int<-69.496 ###intercept

LD.slope<-0.63 ###slope

###number of days gap between failing and renesting

gap<-5

###end of the breeding season

endd<-181

###number of days to wait before trying to remate

wait<-4

###number of days successful nests take to hatch

hatch<-25

###1000 individuals

n.birds<-1000

n.attempts<-4 ###for either 1,2 or 4 possible nesting attempts

###Number of years to run the model for each individuals lifetime.

n.years<-10

### recruitment relationship parameters

recint<-2.27 ###intercept of the recruitment relationship

recslope=-0.04 ###slope of the recruitment relationship

###Set up arrays to store the model output

###time to fail for each attempt

timetofail<-array(time.f$timetofail,dim=c(n.birds,n.attempts,n.years))

###LD - store the birds lay dates even if they go over 181 days (hence extra n.attempts column needed)

LD<-array(NA,dim=c(n.birds,n.attempts+1,n.years))

###LD2 - a dummy lay date array

LD2<-array(NA,dim=c(n.birds,n.attempts+1,n.years))

###success - whether the attempt is successful or not

success<-array(NA,dim=c(n.birds,n.attempts,n.years))

###arrival.mat - arrival dates

arrival.mat<-matrix(NA,n.birds,n.years)

###Randomly select arrival days (see SOM 1.1 for details)

arrival.mat<-sort(c(rnorm(700,120,5),rsnorm(300,140,10,100))) ###same arrival dates in all years for each individual.

###Calculate laying dates for the first attempts in all years (will be the same)

LD[,1,] <- (LD.slope* arrival.mat)+LD.int

###If laying date is > 181 then laying data = NA

LD[,1,]<-ifelse(LD[,1,]<=endd,LD[,1,],NA)

###For the next section the laying dates are scaled so 1 = the first LD

minLD<-min(LD[,1,],na.rm = T)

LD[,1,]<-(LD[,1,]-minLD)+1

endd<-endd-minLD ###scale end

###Calculate success of nesting attempts and the laying dates of the re-nesting events

# laying dates and success

for (y in 1:n.years){ ###for each year

for(i in 1:n.attempts){ ### for each re-nesting attempt

###calculate nest success

success[,i,y]<-ifelse(!is.na(LD[,i,y]),rbinom(n.birds,1,constant),NA)

### Laying dates of re-nesting attempts (save in dummy array)

LD2[,i+1,y]<-ifelse(success[,i,y]==1,NA,(LD[,i,y]+timetofail[,i,y]+gap))

###add to LD array if lay date of re-nesting attempt is < 181 (endd)

LD[,i+1,y]<-ifelse(LD2[,i+1,y]<=endd,LD2[,i+1,y],NA)

} ###close n.attempt loop

} ###close n.years loop

###Calculate mean annual number of breeding attempts

attempts1<-ifelse(!is.na(success),1,NA)

attempts2<-matrix(NA,n.birds,n.years)

for (i in 1:n.birds){

attempts2[i,]<-colSums(attempts1[i,,],na.rm=T)

}

mean.attempts<-rowMeans(attempts2)

###Calculate recruitment

###Remove attempts past day 181 in LD

LD<-LD[,1:4,]

LDsuccess<-success*LD ###Laying date of successful nests only

###Calculate laying date of successful nests in each year

LDsuccessV<-matrix(NA,nrow=1000,ncol=10)

for (k in 1:n.birds){

if(dim(LDsuccess)[2]==1){LDsuccessV[k,]<-LDsuccess[k,,]}

if(dim(LDsuccess)[2]!=1){ LDsuccessV[k,]<-colSums(LDsuccess[k,,],na.rm=T)} ###this returns the laying date for each year of the successful attempt (the colSums isn't really a sum just a way to get the non NA or 0 value)

}###close n.bird loop

LDsuccessV<-ifelse(LDsuccessV==0,NA,LDsuccessV) ###Remove 0s

LDsuccessi<-rowMeans(LDsuccessV,na.rm=T) ###Calculate mean annual laying date for each individual

HDsuccess<-LDsuccessV+hatch ###Calculate the hatch day of the successful nests

###Calculate the mean recruitment probability

recruit<-ilogit((HDsuccess*recslope)+recint)

###Calculate the mean annual recruitment probability of each individual

rec<-rowMeans(recruit,na.rm=T)

###Binomial realisation of recruitment probability

recruit.trail<-matrix(NA,1000,10) ###dataframe to store recruitment realisations

for (ss in 1:1000){ ###for each individual

for (pp in 1:10){ ###for each year

recruit.trail[ss,pp]<-rbinom(1,1,recruit[ss,pp])

}}

###Calculate the total lifetime number of recruits

recruitLT<-rowSums(recuit.trail,na.rm=T) ###total lifetime number of recruits

###Bring all data together

###arrival.mat = arrival dates

###LDsuccessi = Mean annual lay date of successful nesting attempts

###mean.attempts = mean number of breeding attempts

###rec= mean recruitment probability

###recruitLT = total lifetime number of recruits

sim.results=data.frame(arrival=arrival.mat,LDsuccess=LDsuccessi,attempts=4,n.attempts=mean.attempts,recruit.prob=rec,recruit.life=recruitLT)

**References**

Alves, J.A., Gunnarsson, T.G., Sutherland, W.J., Potts, P.M. & Gill, J.A. (2019) Linking warming effects on phenology and demography with range expansion in a migratory bird population. *Ecology and Evolution.*

Þórisson, B. (2013) Farhættir og lýðfræði sandlóu *Charadrius hiaticula*, meistararitgerð, Unpublished MSc thesis, University of Iceland
